# Tissue evacuated during joint replacement procedure as a source of mononuclear cells

**DOI:** 10.1007/s00590-017-2067-9

**Published:** 2017-11-01

**Authors:** Jakobsons Eriks, Erglis Kristaps, Patetko Liene, Erglis Martins, Rasma Dortane, Beatrise Rupaine, Simona Krapse, Briede Ieva, Valdis Goncars, Muiznieks Indrikis, Erglis Andrejs

**Affiliations:** 1 Latvian Centre of Cardiology, Pauls Stradins Clinical University Hospital, Riga, Latvia; 2 Cell Transplantation Centre, Pauls Stradins Clinical University Hospital, Riga, Latvia; 3 Scientific Institute of Cardiology and Regenerative Medicine, University of Latvia, Riga, Latvia; 4 Division of Microbiology and Biotechnology, Department of Biology, University of Latvia, Riga, Latvia; 5 Hospital of Traumatology and Orthopaedics, Riga, Latvia

**Keywords:** Bone marrow, Mononuclear cells, Cell yield, Cell extraction, Cell count, Joint replacement, Excised tissue

## Abstract

**Background:**

Different cell populations from bone marrow were used in various clinical trials for cardiac diseases during last decade. Four clinical studies are ongoing in our institution and enroll patients with cardiac diseases, coronary disease, type 2 diabetes, and osteoarthritis. The density gradient is used to separate bone marrow mononuclear cells. Joint replacement procedures were associated with significant loss of tissue. Usually, excess tissue as bone marrow, peripheral blood and fat are removed to clean operation site. The aim of this study is to prove whether removed tissue during joint replacement procedure can be considered as a significant source of mononuclear cells.

**Methods:**

Excised tissue obtained during joint replacement procedure was collected by AutoLog system. Bone marrow tissue was collected by iliac crest puncture. Mononuclear cells from both sources were isolated by using Ficoll density gradient centrifugation. Flow cytometry was used to detect mononuclear cell, CD34+ population counts and cell viability. Tissue processing yields between the group of joint replacement and iliac crest puncture group were compared.

**Results:**

Together, 34 bone marrow tissue processings were performed. On average, samples contained 46.31 ± 9.35 ml of bone marrow solution. Average cell yield in final product was 28.64 ± 9.35 × 10^6^ MNCs and 0.77 ± 1.51 × 10^6^ CD34+ population. In case of tissue removed during joint replacement nine processings were performed. On average samples contained 450 ± 157.69 ml of tissue solution. Average cell yield in final product was 76.67 ± 35.42 × 10^6^ MNCs and 1.33 ± 0.97 × 10^6^ CD34+ population.

**Conclusions:**

Tissue processing analysis shows that tissue removed during joint replacement procedure can be assumed as a significant source of mononuclear cells. Methods used for bone marrow-derived mononuclear cell extraction can be applied to the excess tissue.

## Introduction

Different cell types were evaluated for their regenerative potential and therapeutic applicability for tissue regeneration. Studies are focusing on the use of cells isolated from bone marrow, peripheral blood, skeletal muscle, adipose tissue and umbilical cord, all of which have shown to improve cardiac function in animal models [1]. Among possible cell sources, autologous bone marrow-derived mononuclear cells (BM–MNCs) and their subpopulations are most extensively tested in clinical trials [2–5].

The leading and most efficient method of surgical treatment on late osteoarthritis stages is total joint replacements. Joint replacement procedures are related to significant loss of tissue on site of operation. A mixture of tissues collected during procedure consists of red bone marrow, peripheral blood, fat, bone debris and rinsing fluid. Usually, the mixture of excised tissue is removed to waste container during rinsing and cleaning of the operation site. So far, no publically available information is present on possible excised tissue use as a mononuclear tissue source.

One of most frequently raised questions related to autologous mononuclear cell application is: Do cell counts matter? From the perspective of autologous application of bone marrow-derived mononuclear cells, therapy could be limited due to variable cell yields. Cell yields vary between individual patients, and the initial tissue sample was restricted due to the internal lumen of the iliac crest.

Besides this, questions concerning cell transplantation exist, including the exact mechanism of the beneficial effect of cell transplantation, the optimal transport of cells into the target tissue, the type and amount of cells implanted, the timing of transplantation, and the assessment of responsiveness of individual patients to the cell therapy [6].

BM–MNC isolation methods are well established; manual or automated processing is performed to produce cell suspensions for transplantation. Currently, different cell delivery methods are employed in clinical praxis, including intracoronary injection and intramyocardial injection [7]. The questions remaining unanswered yet are an effective dose of transplanted cells and best timing for cell transplantation.

In this study, we compared MNC yields between *iliac crest* puncture and excised tissue groups.

## Materials and methods

### Study design

Between 2012 and 2016 the patients with hip and knee osteoarthritis received autologous bone marrow-derived mononuclear cell (BM–MNC) therapy. The bone marrow mononuclear cells were extracted from tissue obtained during hip replacement procedure (study group) and by iliac crest puncture (control group). Extracted cells were released for clinical application. This study aims to compare two sources of tissue used for cell acquisition.

State Central Medical Ethics Committee has approved the clinical study. All patients provided informed consent for the study according to the Declaration of Helsinki, and all patients voluntarily agreed to participate and signed informed consent forms.

The 43 patients who received autologous bone marrow-derived mononuclear cell therapy were included in the study according to the inclusion and exclusion criteria and demographics outlined in Tables 1 and 2.

**Table 1:** Inclusion/exclusion criteria

Inclusion criteria	Exclusion criteria
Degenerative osteoarthritis of the hip or knee	Age over 75 years. Oncologic diseases. Severe kidney, lungs or liver function disorders
Hip replacement procedure (study group)	Hematologic diseases including anemia and thrombocytopenia. Type 1 diabetes mellitus. Severe effusion, contracture and axial deformities in the knee joint
At least 6 months of persisting OA symptoms (control group)	Septic arthritis or skin disorders. Use of corticosteroids and immunosuppressive agents

**Table 2:** Patient demographics

	Excised tissue group	Iliac crest puncture group
Patients (*N*)	9	34
Average age	64	53
Male (*N*)	4	21
Female (*N*)	5	13

### Excised tissue harvesting during joint replacement procedure

During hip replacement operation, artificial joint substitute is inserted. The bone marrow, peripheral blood and fat are removed to rinse and clean operation site prior to insertion of joint substitute. Excised tissue was harvested by AutoLog autotransfusion system (Medtronic) under general anesthesia. Up to 300 ml of liquid phase tissue was aspirated into pre-rinsed AutoLog hardshell reservoir. Operation site was rinsed periodically with sterile 0.9% NaCl (BBraun) containing heparin (500 U/ml). The total volume of heparin and 0.9% NaCl solution used for rinsing was up to 200 ml per tissue harvesting. Solid tissue and bone debris were removed in the waste reservoir. The tissue sample was shipped at room temperature to the central cell processing laboratory and further processed under good manufacturing practice. In short, aspirate was filtrated through 100-µm cell strainer (BD Biosciences) and mononuclear cells were isolated and enriched by density gradient with the use of Ficoll–Paque Premium (GE Healthcare Ltd.) according to manufacturer’s instructions, with minor protocol modifications.

Tissue processing in both patient groups was performed by two biotechnologists to minimize the risks of faults and ensure minimal processing time. All activities, timing, used reagents, batch numbers, shelf-life and personnel involved were recorded to tissue processing file. Each processing is documented in detail and is coded with a particular identifier to ensure processing traceability. All of the processed tissue was released for a therapeutic application.

### Bone marrow harvesting and cell preparation

Bone marrow mononuclear cells were harvested by iliac crest puncture performed under local anesthesia. For adult patients, a 38–45 ml of bone marrow was aspirated into heparin pre-filled syringes (500 U/ml of bone marrow aspirate). The bone marrow aspirate was shipped at room temperature to the central cell processing laboratory and further processed under good manufacturing practice. Aspirate was diluted with sterile 0.9% NaCl (1:5) (BBraun) and filtrated through 100-µm cell strainer (BD Biosciences), and bone marrow-derived mononuclear cells (BM–MNCs) were isolated and enriched by density gradient with the use of Ficoll–Paque Premium (GE Healthcare Ltd.) according to manufacturer’s instructions, with minor protocol modifications.

### Flow cytometry

Samples of the final product were taken and used for flow cytometry analysis within 2 h after processing. Stem-Kit from Beckman Coulter was used for cell labeling with CD45-FITC, CD34-PE, 7-AAD and Stem-Count fluorospheres. Cells were analyzed using FC-500 (Beckman Coulter). Analysis protocol was developed manually. StemCXP program was used for MNC count, CD34+ cell count and cell viability detection. Gating was performed according to ISHAGE protocol according to manufacturer’s suggestions. Cell viability was obtained using the 7-AAD method that is included in ISHAGE protocol [8]. Each measurement contained at least 50,000 events. The maximum number of events was 100,000. Obtained numbers of cells/µl were calculated for the total number of MNCs and CD34+ cells within transplantation material. Measurements with less than 50,000 events were excluded from statistical analysis.

## Results

Bone marrow-derived mononuclear cells were harvested by iliac crest puncture performed under local anesthesia. On average samples contained 46.31 ml of bone marrow solution. Together, 34 BM tissue processings were performed. Same standard operation procedures for all processings were applied. Each final cell solution was released for clinical application. Final product cell counts were analyzed by flow cytometry (see Table 3).

**Table 3:** Cell counts—iliac crest puncture group after processing

	Value	SD
Average sample volume (ml)	46.31	9.35
Average MNC yield (×10^6^ cells)	28.64	31.35
Average CD34+ cell yield (×10^6^ cells)	0.77	1.51

Tissue removed during joint replacement procedure was harvested by AutoLog system under general anesthesia. Filtrating components of a system designed for blood salvage were removed. Reservoir and suction system was used to collect rinsing solution and tissue. On average samples contained 450 ml of tissue solution. Together, nine excess tissue processings were performed. Same standard operation procedures for all processings were applied. Each final cell solution was released for clinical application. Final product cell counts were analyzed by flow cytometry (see Table 4).

**Table 4:** Cell counts—excised tissue group after processing

	Value	SD
Average sample volume (ml)	450	157.69
Average MNC yield (×10^6^ cells)	76.67	35.42
Average CD34+ cell yield (×10^6^ cells)	1.33	0.97

Together, 34 bone marrow tissue processings were performed. On average samples contained 46.31 ± 9.35 ml of bone marrow solution. Average cell yield in final product was 28.64 ± 9.35 × 10^6^ MNCs and 0.77 ± 1.51 × 10^6^ CD34+ population.

In case of tissue removed during joint replacement nine processings were performed. On average samples contained 450 ± 157.69 ml of tissue solution. Average cell yield in final product was 76.67 ± 35.42 × 10^6^ MNCs and 1.33 ± 0.97 × 10^6^ CD34+ population.

## Discussion

The isolation of the mononuclear cell fraction is associated with cell loss. Some studies investigating human bone marrow processing show MNC recovery rates between 15 and 30% after Ficoll density gradient centrifugation [9]. Processing has a significant impact on the cell counts, viability and functional activity of bone marrow-derived progenitor cells. The assessment of cell counts and viability may not entirely reflect the functional quality of cells in clinical application. Some groups suggested that controversial clinical effects in large-scale clinical trials are due to technological differences in cell processing and MNC composition [10]. In fact, it has been proven that efficacy and functionality of MNCs are significantly influenced by red blood cell contamination, the content of apoptotic cells, differences in washing steps and even the centrifugation speed. In this study we focus on bone marrow-derived mononuclear cell and excised tissue-derived cell yields after tissue processing, performed using the same methodology for all patients.

**Fig. 1:**
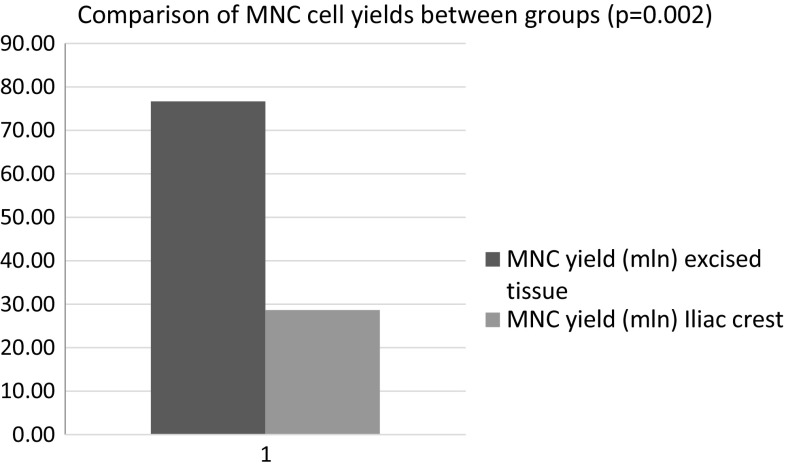
Comparison of MNC cell yields between groups

**Fig. 2:**
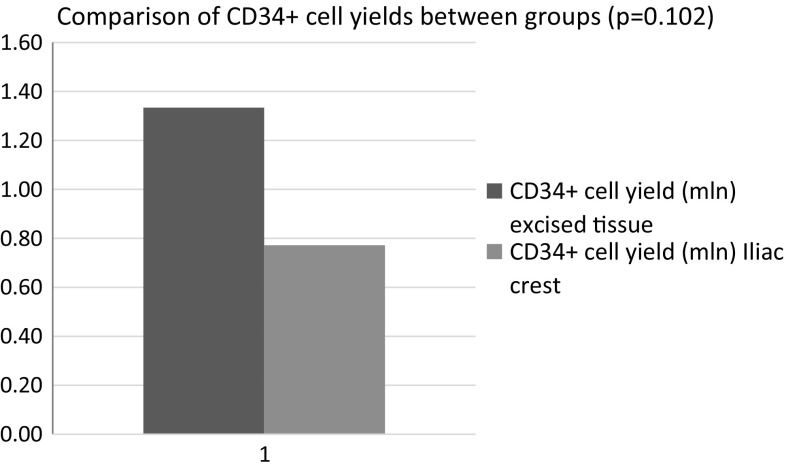
Comparison of CD34+ cell yields between groups

Factors that could affect the cell processing outcome associated with bone marrow extraction are as follows: instruments used—aspiration needle specification (side holes, diameter) and aspiration needle placement (depth, angle, radio control used)—the applied negative pressure, administration of anticoagulation agents, peripheral blood volume in the sample, and the individual patient specificities—iliac crest thickness and internal lumens (vary depending on age, gender and health conditions). In case of excised tissue group main challenges were related to administration of anticoagulation agents—rinsing of surfaces with anticoagulating agent solution is essential to minimize clotting that can lead to lower cell counts in the final product. Preliminary trials show that the presence of fat tissue in processing samples can significantly lower cell yields. Increased application of rinsing solution can lead to higher processing losses and expenses related to materials and consumables. Excised tissue group average MNC extraction yield is 2.71 times higher (*p* = 0.002) comparing to *iliac crest* puncture group, see Figs. 1 and 2. In case of CD34+ cell fraction statistically significant differences in cell yields were not detected. This finding could be associated with asymmetric processing losses (fat contamination, blood clotting) and/or patient age in excised tissue group.

## Conclusions

Tissue processing analysis shows that tissue removed during joint replacement procedure can be assumed as a significant source of mononuclear cells. Methods used for bone marrow-derived mononuclear cell extraction can be applied to the excised tissue. Detailed instructions of harvesting of excised tissue should be developed to minimize fat contamination and minimize volume of rinsing solution in a harvested tissue sample.

## Abbreviations

BM: Bone marrow
MNC: Mononuclear cell
